# Cardiotoxicity of the diamide insecticide chlorantraniliprole in the intact heart and in isolated cardiomyocytes from the honey bee

**DOI:** 10.1038/s41598-024-65007-2

**Published:** 2024-06-28

**Authors:** Mahira Kaabeche, Mercedes Charreton, Aklesso Kadala, Jérôme Mutterer, Pierre Charnet, Claude Collet

**Affiliations:** 1 Institut National de la Recherche pour l’Agriculture, l’Alimentation et l’Environnement, INRAE, UR406 Abeilles et Environnement, Avignon, France; 2 CNRS, UPR 2357, Institut de biologie moleculaire des plantes, 67084 Strasbourg, France; 3 CNRS, UMR 5247, Institut des Biomolécules Max Mousseron, Université Montpellier, Montpellier, France

**Keywords:** Honey bee, Heart, Cardiomyocytes, Electrophysiology, Patch-clamp, Cardiotoxicity, Insecticide, Anthranilic diamide, Chlorantraniliprole, Intracellular calcium channels, Voltage-gated calcium channels, Ryanodine receptors, Cardiovascular biology, Environmental impact

## Abstract

In honey bees, circulation of blood (hemolymph) is driven by the peristaltic contraction of the heart vessel located in the dorsal part of the abdomen. Chlorantraniliprole (CHL) is an insecticide of the anthranilic diamide class which main mode of action is to alter the function of intracellular Ca^2+^ release channels (known as RyRs, for ryanodine receptors). In the honey bee, it was recently found to be more toxic when applied on the dorsal part of the abdomen, suggesting a direct cardiotoxicity. In the present study, a short-term exposure of semi-isolated bee hearts to CHL (0.1–10 µM) induces alterations of cardiac contraction. These alterations range from a slow-down of systole and diastole kinetics, to bradycardia and cardiac arrest. The bees heart wall is made of a single layer of semi-circular cardiomyocytes arranged concentrically all along the long axis of tube lumen. Since the heart tube is suspended to the cuticle through long tubular muscles fibers (so-called alary muscle cells), the CHL effects in *ex-vivo* heart preparations could result from the modulation of RyRs present in these skeletal muscle fibers as well as cardiomyocytes RyRs themselves. In order to specifically assess effects of CHL on cardiomyocytes, for the first time, intact heart cells were enzymatically dissociated from bees. Exposure of cardiomyocytes to CHL induces an increase in cytoplasmic calcium, cell contraction at the highest concentrations and depletion of intracellular stores. Electrophysiological properties of isolated cardiomyocytes were described, with a focus on voltage-gated Ca^2+^ channels responsible for the cardiac action potentials depolarization phase. Two types of Ca^2+^ currents were measured under voltage-clamp. Exposure to CHL was accompanied by a decrease in voltage-activated Ca^2+^ currents densities. Altogether, these results show that chlorantraniliprole can cause cardiac defects in honey bees.

## Introduction

Ion channels are macromolecular complexes inserted in membranes of all cell types, providing routes for ionic fluxes (sodium, potassium, calcium, chloride, etc.) that in turn underlie variations in ionic concentrations in adjacent cell compartments. Owing to their vital role, ion channels have been targeted by agrochemical companies to design neurotoxic and myotoxic insecticides in order to protect crops from insects considered as pests. These compounds have side effects on beneficial insects as well. The deleterious effects of several major classes of insecticides on honey bee ion channels have been previously well characterized^1–13^. These new in vitro data contribute to better anticipate the danger of insecticides towards global insect richness and diversity which have been recently shown to decline drastically, with dramatic consequences to come if strong actions are not taken^14,15^.

Anthranilic diamides are new synthetic insecticides approved in Europe in 2014, first introduced in the French pesticide market and progressively introduced in several other countries such as Germany, Netherlands, Hungary, Romania, etc.^16^. Their toxicity has been ascribed to a capacity to anarchically open a type of intracellular calcium release channels, well known under the name of ryanodine receptors, or RyRs^17^. The use of diamides is presented by agrochemical companies as a credible alternative to major insecticide classes, owing to their original molecular mode of action, their translaminar mobility into leaves and their systemic activity^17^. According to the Eurostat database, chlorantraniliprole (CHL), the first marketed substance of this class, was actually significantly sold in Europe from 2016 onwards (0.7 tons of active substance) and in 2020, anthranilic diamides sales (active substances) had reached 56 tons. In 2022, anthranilic diamides sales reached 28, 12 and 4 tons in Germany, Poland and Netherlands, respectively^16^. The recommended field application rate for CHL initially ranged from 42 to 63 g of active substance per hectare, in the same order as field rates used for pyrethroid insecticides, another widely used class^18,19^. Higher rates of CHL were also recommended in the field afterwards^20^. At national levels, official databases also describe quantities of active substance sold annually, for instance in France, although spatiotemporal descriptions of the actual use in the field are not available yet^21^. In France, the national database on insecticides sales describes significant sales of anthranilic diamides already starting in 2011 (4 tons of active substance) up to 25 tons in 2018^21^. French sales of diamides remained so far well below sales of some other classes (22, 427 and 466 tons of diamides, pyrethroids and organophosphates were sold in 2020, respectively). But the recent ban of neonicotinoid insecticides in the European Union (neonicotinoid sales in France reached 413 tons in 2017 but were drastically reduced in 2020 down to 1.5 tons) and the withdrawal of several other active substances could mechanically boost the use of diamides in the future^21^. According to a recent review based on a market study, diamides accounted for 13% of the 2018 world insecticides sales (in US dollars), i.e. leading to higher financial profits than organophosphates (8%), but lower than pyrethroids (16%) and neonicotinoids (25%)^22^. So far in France, only the active substances chlorantraniliprole (coragen^©^, pagen^©^, as well as 15 other commercial formulations) and cyantraniliprole (Benevia^©^, Mainspring^©^, Verimark^©^) are authorized^23^. A third anthranilic diamide, cyclaniliprole, has been submitted for European approval, but was eventually withdrawn in 2016^24^. Finally, flubendiamide, a phthalic acid diamide with a similar mode of action as CHL hasn’t been granted authorization in France yet, although it is approved at the European level^24^. Interestingly, the Environmental Protection Agency has recommended a withdrawal of flubendiamide from the USA market in 2016^25^.

We have recently studied anthranilic diamides and phthalic acid diamides deleterious effects on ion channels from skeletal muscle and neurons from the bee^8,9^. Our results led us to suspect a high cardiotoxicity of CHL for bees. To date, ion channels involved in the honey bee cardiac function have not been studied in detail, although action potentials (AP) were recorded with intracellular electrodes by Theophilidis and collaborators^26–28^. This group also extensively studied the properties and modulation of bee cardiac force and rhythmicity with mechanotransducers. In other insects however, the properties and pharmacology of ionic fluxes and ion channels underlying the heart myogenic function have been studied using electrophysiology, force measurement and imaging techniques, using ionic substitution and ion channels blockers^29–33^. Mutant flies were also useful in identifying and determining roles of major ion channels responsible for cellular excitability, for instance confirming that *para* voltage-gated sodium channels (Na_V_) were not involved in heart excitability, and showing the importance of potassium channels encoded by the *slowpoke* gene in heart function^34,35^. RNA interference was also useful in suggesting the role of voltage-gated calcium channels (Ca_V_) in fly cardiac excitability^33^. Finally, excitation–contraction coupling and the involvement of intracellular calcium channels has been much less studied than in vertebrates, but insects heart cells do possess dyads and peripheral couplings and thus ultrastructural basis for a functional coupling in between plasma membrane Ca_V_s and intracellular calcium channels^36,37^. A mutation in the *Ryr* gene that encodes RyRs, slows the flies heart rate^38^ and pharmacological manipulations tend to confirm involvement of RyRs in the mealworm heart function^32^.

In the present study, in order to explore CHL effects on the heart function, we assessed its effects on the autorhythmic activity of the heart and studied its mode of action on isolated cardiomyocytes with in vitro cell electrophysiology and calcium imaging.

## Results

### Effects of chlorantraniliprole on heart contraction

**Figure 1:**
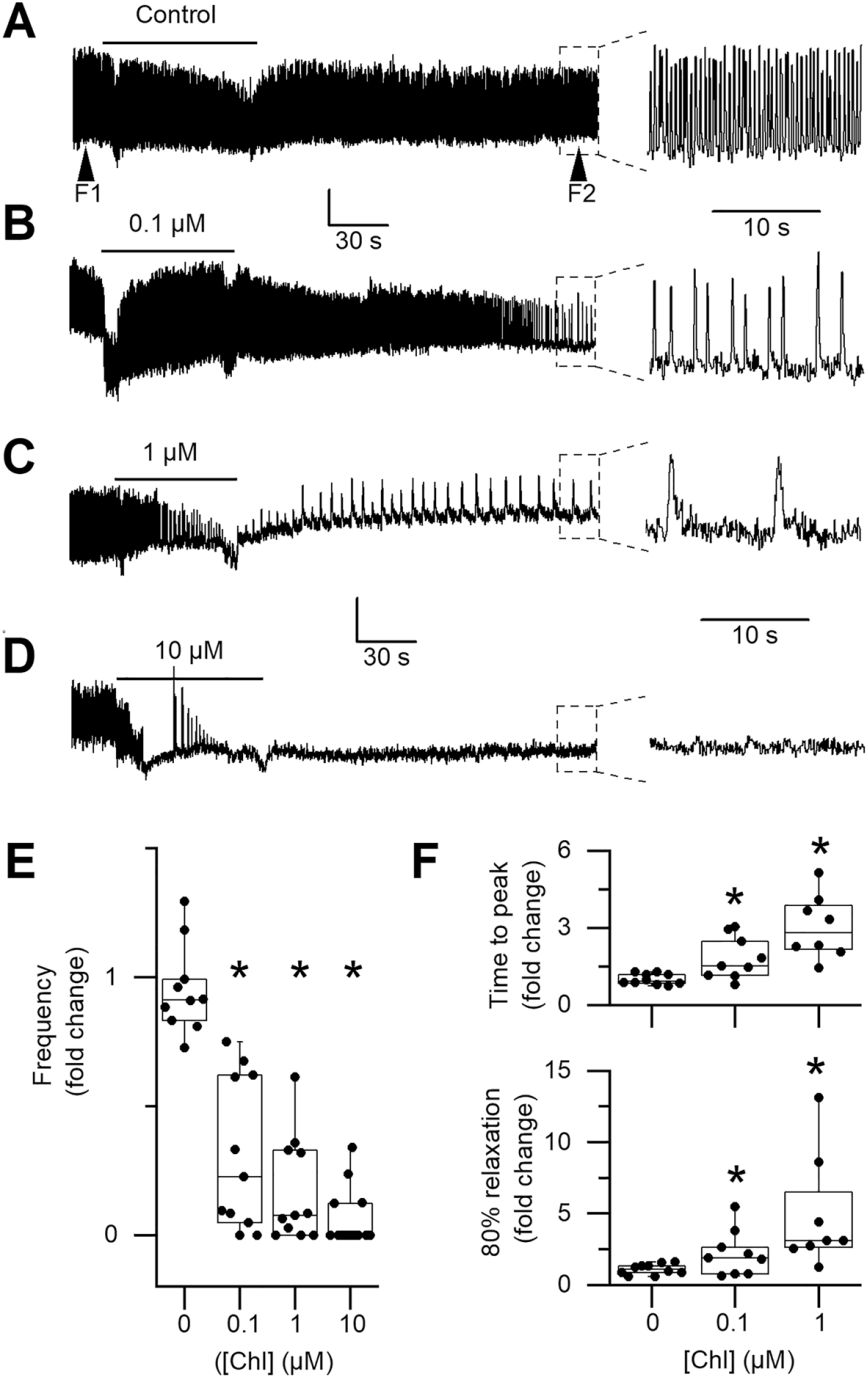
Effects of chlorantraniliprole on heart rate and contraction kinetics. (**A**) The heart rate remains stable after bath exchange with a control solution. Frequency was measured before exchange (left arrowhead, F1) and at the end of the 300 s recording period (right arrowhead, F2). Right: trace expanded to better show the last 20 s of recordings. (**B**–**D**) Decrease in heart rate and changes in contraction kinetics after exposure to 0.1, 1 and 10 µM CHL. In (**D**) an example of cardiac arrest at 10 µM. (**E**) Evolution of frequency was calculated for each heart as F2/F1. The frequency at the end of recording was thus expressed as a fraction of the heart initial rate. Cardiac arrest was more frequently observed at 10 µM (‘0 values’). All CHL concentrations statistically decrease heart rate. (**F**) CHL statistically slows both the contraction and the relaxation phases. Boxplots show individual data and quantiles. Vertical scale bars (**A**–**D**): 5 A.U. Asterisks indicate significant differences (paired Wilcoxon test).

A total of 45 hearts from adult bees were dissected in order to explore the effects of chlorantraniliprole ex vivo. Before exposure, in Tyrode solution, the heart rate was on average 3.4 ± 0.2 Hz (n = 45). A total of 10, 11, 11 and 13 hearts were exposed to CHL concentrations of 0, 0.1, 1 and 10 µM, respectively. Figure 1A shows a typical heart activity recorded during a control solution exchange. The contraction rate of this heart remained stable and turned from 2.6 Hz before exchange to 2.5 Hz at the end of the recording (the right part of the figure shows a zoom on the last 20 s of recording). To ease comparison from one heart to another and in between CHL concentrations, for each heart, the frequency at the end of the recording (Fig. 1, F2) was normalized to the frequency before bath exchange (Fig. 1, F1). Figure 1E shows that some hearts can undergo either a slight increase or a slight decrease in frequency after bath exchange with the control solution (left box plot, 0 µM CHL). However, on average, frequency was not significantly decreased by the bath exchange procedure (5% decrease on average, n = 10, Wilcoxon paired test p = 0.3223). Note that the boxplot representation was chosen to show individual data distributions and medians. Exposure to the weakest CHL concentration (0.1 µM) induced measurable effects on hearts activity. Figure 1B illustrates a more than twofold decrease in frequency in one heart (from 3.2 Hz before exchange to 1.3 Hz at the end of recording, *i.e.* 3 min after exposure). At this concentration, two hearts out of eleven even underwent a total arrest (see ‘0’ values, Fig. 1E, 0.1 µM). Milder frequency effects were seen at this concentration as well. Various levels of bradychardia were thus observed from one heart to another and overall at 0.1 µM, a ~ threefold statistically significant decrease in frequency was observed (3.7 ± 0.3 Hz before exposure and 1.1 ± 0.3 Hz at the end of the recording, n = 11, Wilcoxon test P = 0.001). At higher CHL concentrations, effects on frequency were exacerbated with one third of hearts (3/11) and two third (9/13) undergoing failure, at 1 and 10 µM, respectively (see ‘0’ values in Fig. 1E and an example of heart failure at 10 µM, Fig. 1D). On average, heart rate thus decreased 6- and 17-fold at 1 and 10 µM, respectively (from 3.4 ± 0.4 Hz to 0.6 ± 0.2 Hz at 1 µM, n = 11 and from 3.4 ± 0.3 to 0.2 ± 0.1 Hz at 10 µM, n = 13, Wilcoxon test, P = 0.001 and 0.0002). A Kruskall-Wallis Anova was performed on normalized data (Fig. 1E) confirming that CHL had an effect on heart frequency (F_3,45_ = 27.38, p < 0.0001). A post-hoc Dunn’s test shows that all CHL concentrations induce a significant decrease in frequencies as compared with control (p < 0.05). The bradycardia observed at 0.1 µM was accompanied by alterations in contraction kinetics. First of all, an increase in the duration of the systolic phase was detected by measuring the time to reach the contraction peak (time to peak, TTP). The TTP was calculated for each heart from 5 consecutive contractions measured before exposure and 5 consecutive contractions measured 3 min after exposure. At 0.1 µM, TTP increased ~ twofold (Fig. 1F upper panel, 0.12 ± 0.01 s to 0.22 ± 0.04 s, n = 9, Wilcoxon test P = 0.0195). In control conditions, no such effect was observed, with a mean fold change of 1.01 ± 0.07 (on average, TTP was 0.13 ± 0.01 s both before and after solution exchange, n = 10, Wilcoxon P = 0.9102). In addition to heart frequency changes, strong contraction kinetics changes were also observed at 1 µM, with a TTP increase threefold (from 0.11 ± 0.01 to 0.33 ± 0.07 s, n = 8, Wilcoxon test, P = 0.0156). A Kruskall-Wallis Anova was performed on TTP normalized data (Fig. 1F, upper panel) confirming CHL has an effect on the systolic phase (F_2,27_ = 14.81, p < 0.001). A post-hoc Dunn’s test confirms that 1 µM CHL induces a significant TTP increase as compared with control (p < 0.001). At 10 µM, most hearts have stopped beating at the end of recordings and no attempt was made to characterize TTP kinetics after CHL perfusion. Another obvious sort of kinetics change was observed at 1 µM during the diastolic phase, that is a slow-down of the cardiac relaxation phase. As shown in Fig. 1F (lower panel), the time to reach 80% of relaxation significantly increased 3.5-fold at this concentration (from 0.14 ± 0.03 s to 0.47 ± 0.08 s after exposure, n = 8, Wilcoxon test, P = 0.0078). A similar tendency was observed in some hearts exposed to 0.1 µM CHL, but overall the effect was not found statistically significant when paired comparison were made (from 0.13 ± 0.02 s before exposure to 0.26 ± 0.06 s after exposure, n = 9 Wilcoxon test, P = 0.0742). A Kruskall-Wallis Anova followed by post-hoc Dunn’s test was performed on relaxation normalized data (Fig. 1F, lower panel) confirming CHL has an effect on the diastolic phase (F_2,27_ = 11.38, p < 0.01). The post-hoc Dunn’s test confirms that 1 µM CHL induces a significant slow-down of the diastolic phase as compared with control (p < 0.01).

In conclusion of this section, CHL can alter heart function *ex-vivo* at concentrations mimicking realistic doses, *i.e.* doses received in the field (see discussion). In the *ex-vivo* configuration, honey bee heart function does not rely on modulation by the nervous system any more (ventral nerve chord is dissected out). However, whereas the insect heart tube has been demonstrated earlier to have an intrinsic contraction capacity (myogenic contraction, see Discussion), its activity may depend on so-called alary muscles (made of skeletal muscle fibers) that suspend the tube to the abdominal tergites^26^. Alary muscles likely facilitate the diastolic phase by expanding back the heart tube after its contraction. They not only help to refill the heart, inducing a depression inside the tube that drives entry of the hemolymph through ostia, but they also prevent its irreversible collapse. Thus, although CHL effects demonstrated right before could result from direct effects on cardiomyocytes, it could also results from effects on skeletal muscle fibers, as demonstrated by our group earlier^9^. Therefore, in order to explore a direct effect of CHL on cardiomyocytes, an enzymatic cell isolation procedure was developed. This type of procedures is routinely made on vertebrate heart to explore cardiomyocytes physiology, but it has very seldomly been performed on insects and never on the honey bee heart. Young bees were used, since enzymatic isolation of skeletal muscles proved to be much more efficient than with older bees.

### Chlorantraniliprole induces contraction in intact cardiomyocytes

**Figure 2:**
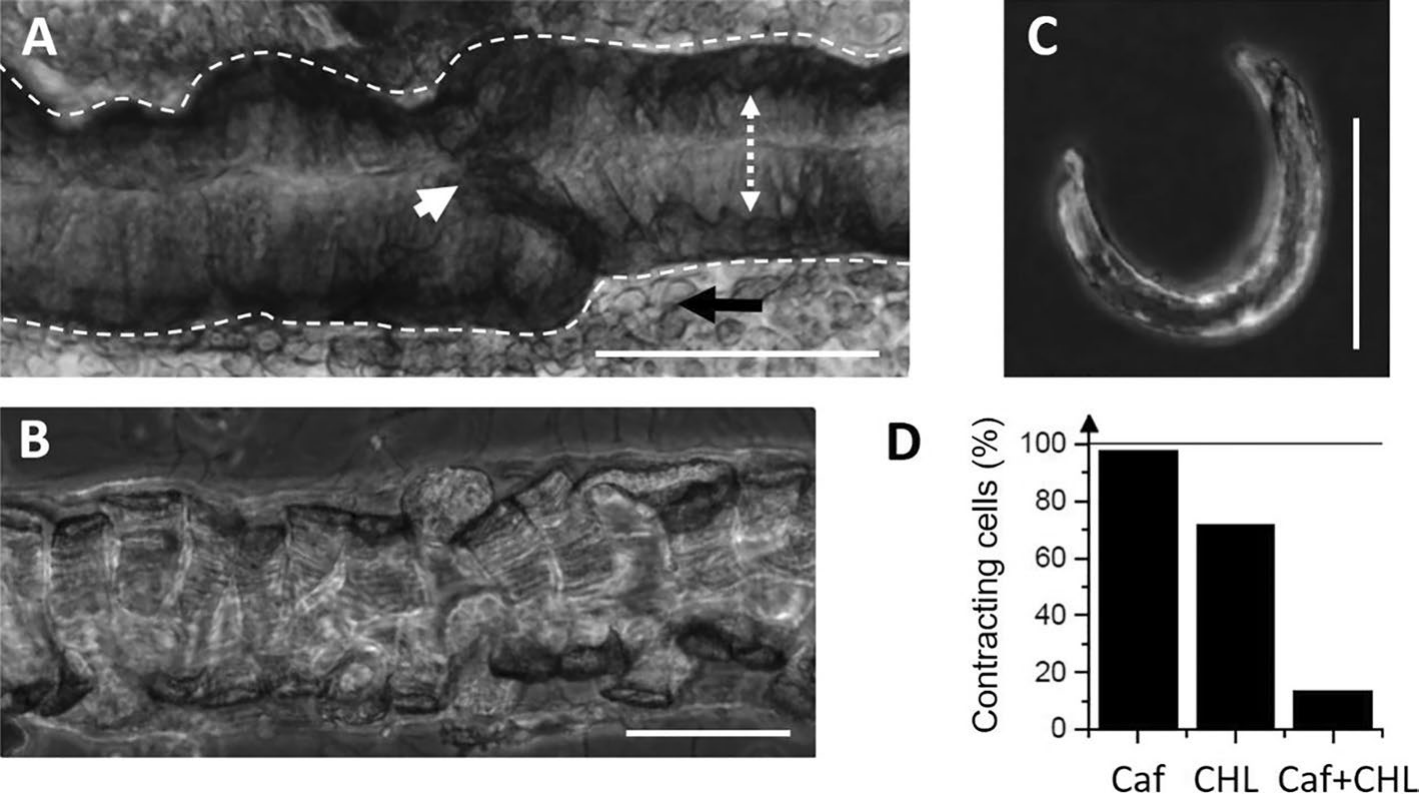
Effect of caffeine and chlorantraniliprole on isolated cardiomyocytes. (**A**) Portion of heart dissected out from a bee after PFA tissue fixation (see text for details regarding picture annotations). (**B**) Portion of a fresh bee heart after dissection and enzymatic digestion. (**C**) A cardiomyocyte isolated after enzymatic and mechanical dissociation, laying on the bottom of a Petri dish. (**D**) In separate experiments, single cell superfusion with caffeine 20 mM (Caf) or CHL 10 µM both induce a strong contraction of cardiomyocytes. Only a small percentage of cells contracted upon caffeine exposure when CHL was applied before (Caf + CHL). Scale bars: 200 µm in panel A and 100 µm in panels B and C.

A portion of a heart dissected out from a bee after tissue fixation (with paraformaldehyde, PFA 4%) and observed under an inverted light microscope is shown in Fig. 2A. The tube width (indicated with a white dashed line) and its lumen (dotted arrow) show width variations along its length. The junction between two adjacent chambers is indicated with a white arrowhead. The heart lays on a translucent membrane named dorsal diaphragm (some cells of the diaphragm are indicated by a black arrow). The wall of the heart tube consists in a single layer of cells forming a series of adjacent concentric rings. Each ring, perpendicular to the heart long axis, is formed by two semi-circular cells facing each other (two opposing rows of myocytes) and attached by their endings.

**Figure 3:**
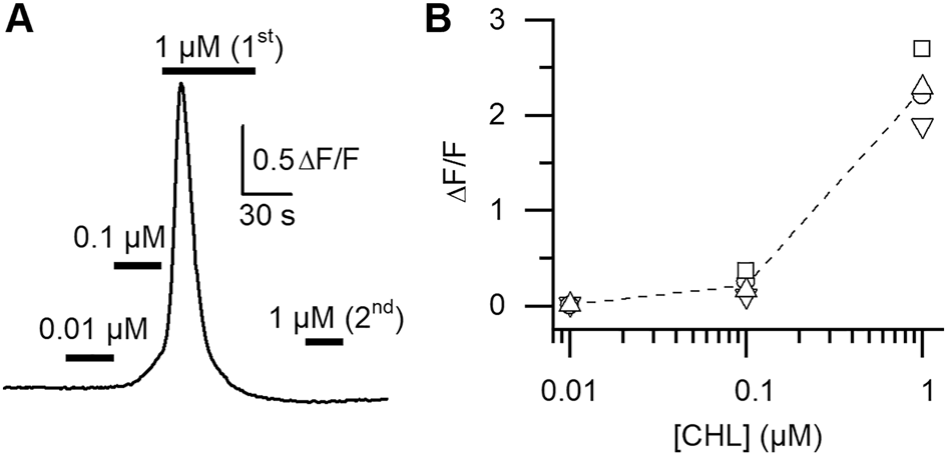
Chlorantraniliprole-induced intracellular [Ca^2+^] variations measured in isolated cardiomyocytes. (**A**) In the absence of external Ca^2+^, CHL induces a concentration-dependent increase in [Ca^2+^]_i_. At 1 µM (1st exposure), [Ca^2+^]_i_ reaches a maximal value in ~ 10 s and decreases back towards the resting level at a similar speed in spite of the continuous perfusion with the molecule. A second exposure to 1 µM (2nd) fails inducing another response. (**B**) Concentration-dependent fluorescence increases obtained in 4 different cells (different symbols). The dashed line links the mean ΔF/F_0_ calculated at each concentration for these 4 cells.

Depending on the heart tube diameter along the abdomen (from 120 to 250 µm), theoretical cells length thus ranges from 220 to 400 µm. In situ, cells appear slightly inflated, with a thickness of ~ 25 µm and a width of ~ 50 µm. After enzymatic dissociation of a fresh heart, several cell types can be dispersed in Petri dishes. Some intact heart portions still enclosed in their pericardial sheath can be observed (Fig. 2B) with intercellular junctions clearly loosen by enzymatic action. A number of isolated cardiomyocytes (~ 20–50 per heart) can be obtained (Fig. 2C), as well as small bundles of 2–3 cardiomyocytes still attached with each other (not illustrated). Other isolated cell types can be identified such as alary muscle fibers, haemocytes, oenocytes, or trophocytes^26,39^. After isolation, intact cardiomyocytes keep the semi-circular shape they had in situ. On average, cardiomyocytes were 282 ± 13 µm long and 25 ± 1 µm thick (n = 78). Their width could be occasionally measured when cells laid on their side opposed to the heart lumen (~ 50 µm, not illustrated). CHL effects on isolated cardiomyocytes were assessed visually under transmitted light microscopy. A majority of cardiomyocytes (72%) exposed few seconds to a gravity-driven perfusion containing CHL 10 µM undergo contraction (Fig. 2D, n = 46). In a second batch of cardiomyocytes, the well-known RyRs activator caffeine (20 mM) induced a strong contraction of 97% of the cells (n = 80). The efficiency of the RyR agonist caffeine suggests that the calcium content in sarcoplasmic reticulum remains sufficient to trigger contraction even after the cell isolation procedure. A third batch of cardiomyocytes were exposed to CHL first and then caffeine. In this case, only 14% of cells responded to caffeine after a preexposure to CHL (n = 44). Most cells exposed first to CHL do not respond to caffeine, an indication that the intracellular Ca^2+^ stores have thus been emptied by CHL, a result consistent with the mode of action of diamides. In order to verify that CHL was able to induce cytoplasmic Ca^2+^ elevations in the absence of extracellular Ca^2+^, Fluo-8 fluorescence signals were measured (Fig. 3). Figure 3A shows a representative cell. Despite a transient intracellular cytoplasmic Ca^2+^ increase ([Ca^2+^]_i_) due to mobilization of intracellular stores, the cell did not contract. After a first exposure to CHL 1 µM (1^st^), a second exposure to CHL 1 µM (2^nd^) no longer induced any fluorescent increase, suggesting that intracellular stores have been emptied by the 1^st^ exposure. Qualitatively similar responses were obtained in 3 other cells. Calcium transients amplitudes were only quantified in cells in the absence of contraction (as strictly required when using a non-ratiometric indicator). Exposure to CHL 0.1 µM and 1 µM led to a mean increase of 0.22 ± 0.06 and 2.28 ± 0.16 ΔF/F_0_, respectively (Fig. 3B, n = 4 cells). In 3 other cells, CHL 1 µM induced contracture of the cell before reaching the maximal fluorescence response, and in this case no attempt was thus made at measuring Ca^2+^ transients amplitudes. The lowest concentration (0.01 µM) did not induce detectable transients.

### Ionic currents in cardiomyocytes and effects of chlorantraniliprole on Ca_V_s

**Figure 4:**
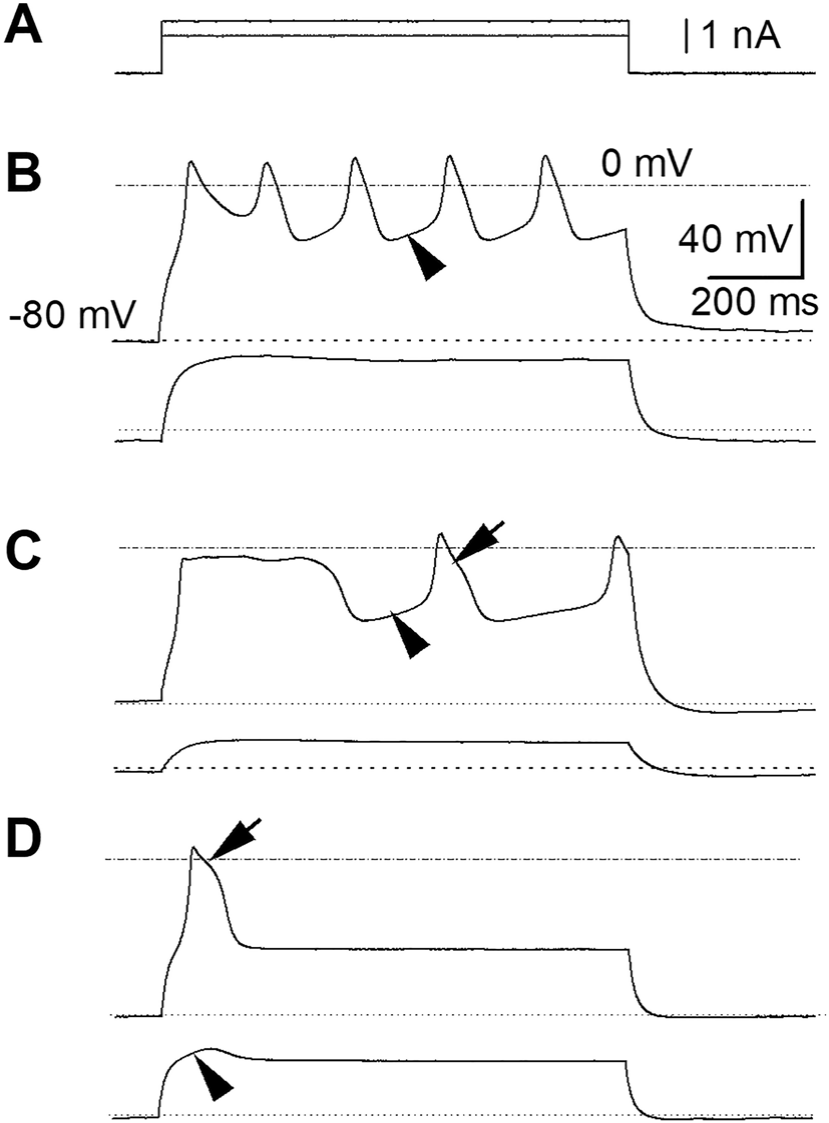
Diversity of action potentials in cardiomyocytes. (**A**) Current steps injected in current-clamp mode. (**B**) Membrane passive electrotonic potential in response to a subthreshold stimulation (lower trace) in one cardiomyocyte. Train of five overshooting action potentials (peak above 0 mV) obtained in response to a suprathreshold stimulation in the same cell (upper trace). Action potentials show a slow depolarization phase (arrowhead). (**C**) In another cardiomyocyte, an electrotonus (lower trace) and two long action potentials are obtained (and a third incomplete PA). The first AP shows a rather long plateau. The second AP shows both a slow depolarization phase (arrowhead) and a plateau (arrow). In a third cell (**D**), a gradual active response superimposes to the passive electrotonic potential (lower trace, arrowhead). In this cell, higher current injection could only trigger a single AP with a plateau, whatever the stimulus intensity (upper trace arrow).

**Figure 5:**
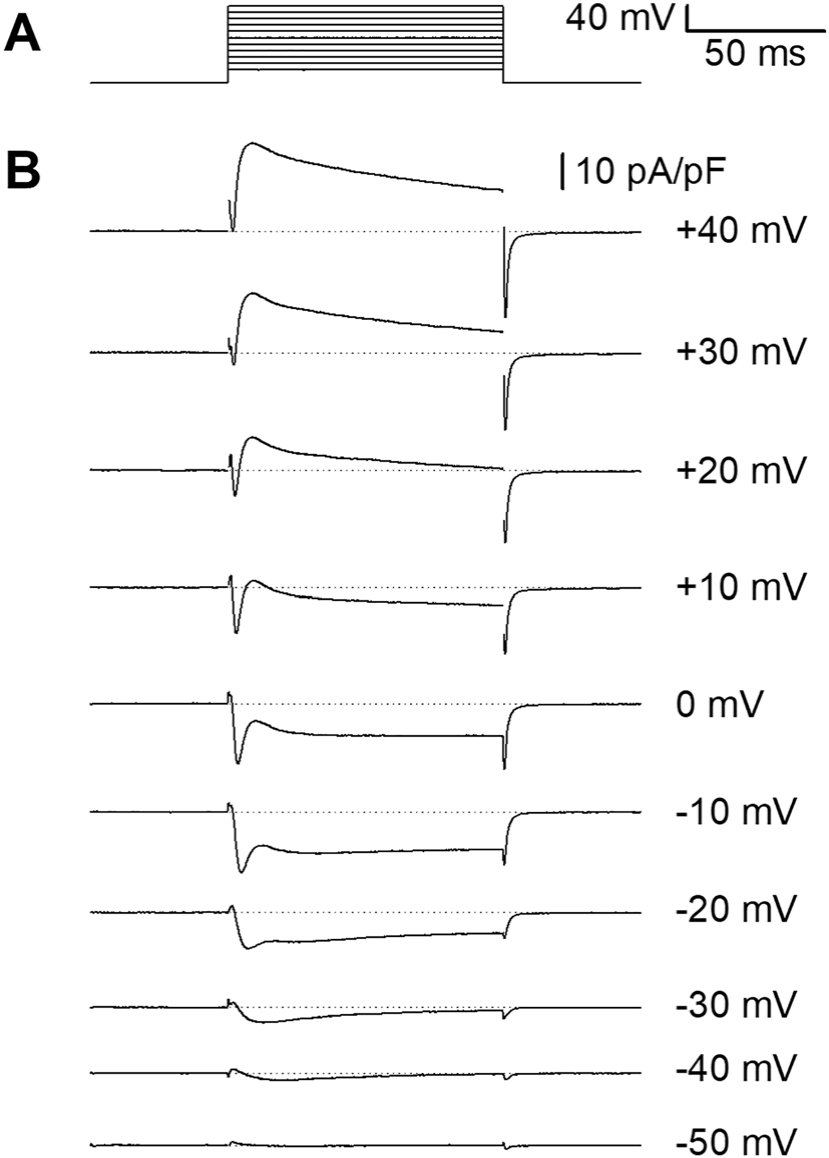
Total voltage-gated ionic currents in cardiomyocytes. Total ionic currents recorded under voltage-clamp in a Tyrode extracellular solution (**B**), in response to a series of 100-ms step depolarizations of increasing amplitude from a holding potential set at − 80 mV (**A**). In this cell, a low-voltage activated inward current is recorded from ~  − 50 mV and a high-voltage activated inward current is recorded from ~  − 30 mV. These two components can be ascribed to CaVs in the absence of NaVs in the insect heart.

In a previous study on bee skeletal muscle fibers CHL-induced disruption of Ca^2+^ homeostasis was accompanied by a decrease in voltage-gated calcium currents^9^. In the present study, a characterization of bee cardiomyocytes electrophysiological properties was performed. It aimed at exploring the possibility that CHL toxicity could be associated with effects on cardiac voltage-gated calcium channels as well. Action potentials were first recorded in the current-clamp mode, with EGTA in the pipette to prevent cytoplasmic calcium increase and with Tyrode as extracellular solution (Fig. 4). An attempt to measure resting membrane potential was first made in the I_0_ mode of the amplifier, and the mean membrane potential was − 48 ± 2 mV (n = 19), a value consistent with earlier measurements made with intracellular glass microelectrodes in *ex-vivo* bee hearts^26,27^ and from the adult moth^36^. A basal current was thus systematically injected to set the resting membrane potential at -80 mV in order to ensure a recovery of voltage-gated channels from voltage inactivation. Cells were submitted to a series of 1-s current injections of increasing amplitude (Fig. 4A). Below the channels’ activation threshold, step current injections induced a passive response of the membrane (Fig. 4B, lower trace). With higher step current injection, all-or none action potentials were recorded (Fig. 4B–D upper traces from 3 different cells). AP consistently repolarized to a membrane potential value close to the resting membrane potential measured in *ex-vivo* hearts in similar ionic conditions^27^. Some AP showed a plateau (Fig. 4C,D, arrow). A slow depolarizing phase (Fig. 4B,C, arrowhead) preceded a faster depolarization phase overshooting 0 mV. Graded voltage responses could also be recorded in some cells (Fig. 4D, arrowhead). In some cells, only a single AP was recorded, whatever the intensity of the suprathreshold injected current (Fig. 4D, upper panel). Under voltage-clamp, inward and outward currents (normalized to the cell capacitance in pA/pF) were activated in response to 100 ms depolarizations of increasing amplitude from a holding potential of -80 mV (Fig. 5). From ~ -50 mV a transient inward component was observed. From -30 mV, a sustained inward component merged with the former component. These two components can be ascribed to the presence of voltage-gated Ca^2+^ currents (see results below) rather than voltage-gated Na^+^ channels, since the latter do not contribute to insect heart action potentials and beating rate^31,32,35^. For higher voltages, outward components activated, as expected from potassium channels expression in insect cardiomyocytes.

**Figure 6:**
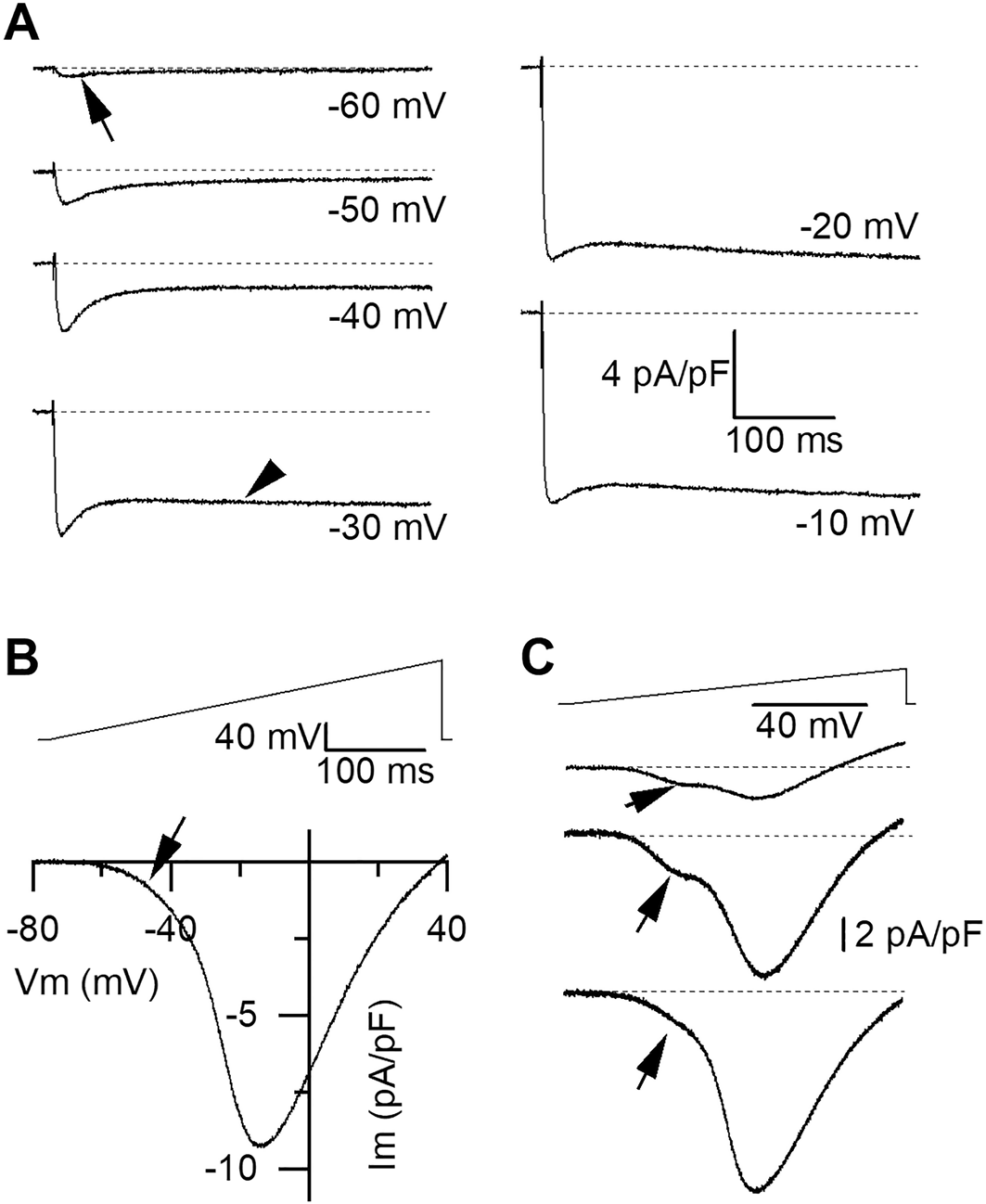
Low and high-voltage activated calcium channels in cardiomyocytes. Voltage-gated currents recorded in an extracellular solution favouring recordings of barium currents through calcium channels. (**A**) Depolarizing 400 ms-long voltage steps from a holding potential set at − 80 mV trigger the opening of voltage-gated calcium channels. From -60 mV, a fast activating and fast-inactivating current (arrow) is observed in this cardiomyocyte. From − 40 to − 30 mV a sustained current (arrowhead) superimposes to the fast component. The maximal current amplitude is reached at − 10 mV. (**B**) In the same cell as A, an I/V curve (lower panel) is obtained in response to a voltage ramp ranging from − 80 to + 40 mV in 400 ms (upper panel). The I/V curve activates from a low voltage (-60 mV), with a foot (arrow) ascribed to the low-voltage activated current seen in A. (**C**) The low-voltage activated component of the I/V curve can be seen in a majority of cells (e.g. from 3 cells).

In order to study cardiomyocytes voltage-gated Ca^2+^ channels, an extracellular solution favoring recordings of barium currents through Ca_V_s was used (see Solutions). In these conditions, two types of currents can be identified in response to step depolarizations from a holding potential set at -80 mV. A current with fast activation and inactivation kinetics activates from a threshold of ~ − 50 to − 60 mV, consistent with the presence of a low-voltage-activated T-type current (Fig. 6A, arrow). At higher potentials (from ~ − 40 mV), a second component of current is observed, with long-lasting kinetics, consistent with a high-voltage activated L-type current. In Fig. 6A, the maximal amplitude of the total barium current was obtained with a step depolarization to − 10 mV (− 8.5 pA/pF). To ease the exploration of Ca_V_s voltage-dependence and pharmacology, protocols of 400 ms voltage-ramp ranging from − 80 mV to + 40 mV (0.3 mV/ms) were used. Figure 6B shows the I/V curve obtained with a ramp in the same cell as in Fig. 6A. The arrow indicates an early activation of a low-voltage activated calcium channels. In this cell, the maximal inward current reached 9 pA/pF at potential − 15 mV and the reversal potential was near + 40 mV. The I/V curve obtained from one cell to another showed variable current amplitudes as illustrated in Fig. 6C, with an average amplitude of − 7.8 ± 1.9 pA/pF (n = 22). Two major types of I/V curves were observed: those with a low-voltage-activated component (e.g. from 3 cells in panel C) and those without it. As shown in panel C, the low-voltage component was more or less prominent as compared with the high-voltage component. Eighty-six percent of cells showed a low-voltage activated component, while no evidence of such component could be observed in three out of 22 cells. Three cells also showed evidence of a low-voltage activated component without clear high-activated component. The mean reversal potential measured on I/V curves was 26 ± 2 mV (n = 22).

**Figure 7:**
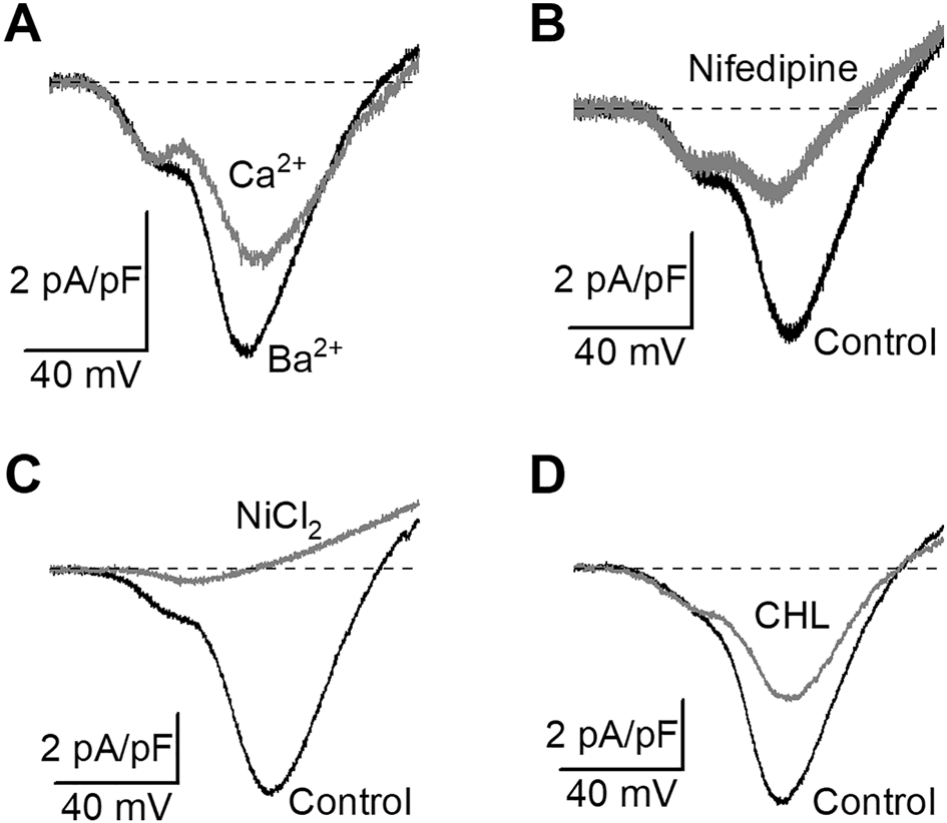
Basic pharmacology of calcium channels and effects of chlorantraniliprole. (**A**) Current–voltage relationship of Ca_V_s with barium or calcium as the divalent charge carrier. (**B**) Effect of nifedipine (10 µM) on Ca_V_s I/V curve. (**C**) Effect of nickel (2 µM). (**D**) Effect of CHL (10 µM).

In the same extracellular solution as previously, when Ca^2+^ was substituted to Ba^2+^ as charge carrier, the current was smaller (Fig. 7A), suggesting that Ba^2+^ permeates better than Ca^2+^ through cardiomyocytes Ca_V_s. On average, the maximal current measured at peak of the I/V curve was 25% smaller with Ca^2+^ than with Ba^2+^ (6 to 46%, n = 7 cells). The peak maximal current density was − 8.6 ± 2.7 and − 6.3 ± 2.0 pA/pF, with Ba^2+^ or Ca^2+^, respectively (n = 7, p < 0.05, Wilcoxon test). An estimation of the total inward charges (in pCb) flowing during the 400-ms voltage ramp was given by integrating the negative part of the curve. This value was normalized to the cell capacitance and expressed in pCb/pF. With Ca^2+^, the total charge density was 15% smaller (− 1.01 ± 0.34 and − 1.20 ± 0.39 pCb/pF with Ca^2+^ and Ba^2+^, respectively, n = 7). In Fig. 7B, after 1 min perfusion with the L-type Ca_V_s blocker nifedipine (10 µM), currents through Ca_V_s decreased, with a major effect seen on the high-voltage activated component (see also supplementary Fig. S1), and no clear effect on the low-voltage activated one. On average, the peak amplitude of the I/V curve decreased by 77 ± 9% (63–95%, n = 3). The total inward charges measured as the integral of the negative part of the curve was on average 75 ± 10% smaller (61–95%, n = 3) with nifedipine (− 0.14 ± 0.05 pC/pF, n = 3) than in control conditions (− 0.89 ± 0.47 pC/pF). In another set of experiments, superfusion with NiCl_2_ 2 µM reduced both the low-activated and the high activated components of currents through Ca_V_s (Fig. 7C). On average, the I/V peak amplitude was reduced by 93 ± 3% (81–100%, n = 5), from − 7.8 ± 0.4 pA/pF to − 0.5 ± 0.3 pA/pF (p < 0.01, paired t-test). The total inward charges measured as the integral of the negative part of the curve was on average 94 ± 3% smaller (81–100%, n = 5) with NiCl_2_ (− 0.06 ± 0.04 pC/pF, n = 5), than in control conditions (− 0.97 ± 0.07 pC/pF). In a last set of experiments, the effect of CHL 10 µM was explored. Figure 7D shows the control I/V curve right before addition and after 2 min superfusion with CHL. The effects were quite variable from one cell to another but on average, the I/V curve peak amplitude was significantly reduced by 47 ± 12% in CHL (5–85%), from − 4.9 ± 3.0 pA/pF in control to − 2.7 ± 1.6 pA/pF in CHL (n = 7, p = 0.01563, paired Wilcoxon rank test). The total inward charges measured as the integral of the negative part of the curve was on average 41 ± 8% smaller (25–82%) with CHL (− 0.37 ± 0.21 pC/pF), than in control conditions (− 0.68 ± 0.42 pC/pF, n = 7). The high-voltage activated current component was the most affected in all cells but the low-voltage activated component was also decreased in 2 out of 7 cells.

## Discussion

The present results demonstrate the cardiotoxic potency of CHL for bees. The decrease in heart beat frequency was accompanied by both an elongation of systolic and diastolic phases. Provided these cardiac alterations also take place in situ, they may contribute to the honey bee locomotor deficits readily described in the initial Draft Assessment Report for CHL^40^. Cardiotoxicity may explain symptoms observed with anthranilic diamides in other insects as well (lethargy, paralysis)^41^. Cardiac effects combine with effects identified earlier both on the skeletal muscular system and the nervous systems of bees^9^. The effects of CHL (0.1–10 µM) on the bee heart appear stronger than in cockroach hearts. Indeed, CHL 200 µM decreased the isolated adult cockroach heart beat frequency by only ~ 20% after 40 min exposure^42^. A strong effect of CHL has also been described in moth larvae (50% decrease in the heart rate after body injection with 30 ng)^43^. Other candidate anthranilic diamides have been identified as potent modulators of the moth larvae heart^41^. This is the case for compound DP-012, which resembles CHL, with some differences in halogens substitution on the pyrazole ring and on the benzyle ring. Thirty minutes after injection of 10 ng/g of body weight with compound DP-012, a strong decrease of the heart rate was measured in moth larvae (-55%). Assuming a volume of 1 ml for these larvae (weight 1 g), and an homogenous distribution in the body, the injected dose 10 ng would correspond to a body concentration of ~ 100 nM^44^. Ten and one 100- fold higher doses of candidate compounds DP-010 and DP-002 were necessary to induce cardiac modulations similar to DP-012, respectively (− 63% and − 52%)^41^.

CHL anarchically mobilizes Ca^2+^ from the calcium stores in honey bee skeletal muscle fibers^9^. CHL also stimulates Ca^2+^ release from cockroach neurons as demonstrated early by the chemists who designed the compound^45^. Flubendiamide, another diamide insecticide has similar effects in honey bee olfactory neurons from the antennae^8^. In order to know whether the alterations of bee cardiac contractility were in part due to direct alteration of heart cardiomyocytes, we have isolated cells from the heart tube. This procedure is quite traditional to study vertebrate heart cells physiology but to our knowledge, it has only seldomly been performed in insects^33^. As also shown earlier in skeletal muscle fibers, CHL was able to induce intracellular Ca^2+^ transients and contraction of isolated cardiac myocytes. Intracellular Ca^2+^ transients can be triggered in the absence of extracellular Ca^2+^, confirming the involvement of internal stores in CHL-induced signals. After exposure to CHL, responses to caffeine were decreased, suggesting that internal stores were emptied by CHL. After exposure to CHL 1 µM, a second challenge with CHL could no longer induce Ca^2+^ transients. Another untested possibility would be that exposure to CHL inhibits the contractile machinery, but to our knowledge, this effect has never been reported. CHL has been described as selective RyR activator^46^, but in bee cardiac myocytes, exposure to CHL also leads to a decrease of currents amplitudes through Ca_V_s, confirming the effect earlier described in skeletal muscle fibers and neurons^9^. We have measured two types of calcium currents with distinct activation thresholds in bee cardiomyocytes. Their voltage-dependence, kinetics and basic pharmacology suggest they match the two types of calcium currents that we have already identified in bee skeletal muscle fibers^47–49^. In skeletal muscles, their kinetics and pharmacology led us to relate these currents to the expression of Ca_V_1 and Ca_V_3 channels, only one gene of each being expressed in bees^50^. Fly cardiomyocytes express Ca_V_1 (Ca-α1D) and Ca_V_3 (Ca-α1T) channels as well^33^. In the absence of voltage-gated sodium channels in insect heart cells^30,31^, both Ca_V_s participate to the cardiac action potential depolarization phase. The high-voltage activated component recorded in cardiomyocytes is sensitive to nifedipine, as also shown in skeletal muscle cells or neurons^48,51^. A nifedipine-sensitive component was also demonstrated in fly cardiomyocytes^33^. A more extensive pharmacology of Ca_V1_ and Ca_V3_ could be performed in the future with electrophysiological protocols aiming at separating currents flowing through these channels.

The shape of action potentials recorded here with a patch-clamp pipette matches pace-maker-like action potentials recorded previously with fine intracellular microelectrodes in the bee heart^26^. Both Ca^2+^ current components that we have identified may be involved in generating the pacemaker autorhythmic peristaltic activity of the bee heart. As previously shown in other insects, the contraction of the heart tube is indeed myogenic, *i.e.* it’s not triggered by nerve impulses and unlike in vertebrates, the pacemaker activity is not localized to a specific area of the insect heart^29,52^. A diversity of AP kinetics were observed in bee cardiomyocytes, as also shown in the heart of the adult moth^29^. In the insect heart, action potentials shapes can be of graded nature, as also shown in insect skeletal muscle^36,48^. The graded nature of APs may allow for a finer tuning of the muscle contraction strength with a gradual recruitment of cells involved. Simultaneous recording of contraction and action potential showed that the slow initial depolarization phase accompanies diastole^27^, that is the heart relaxation. The diastolic depolarization may be underlained by low-voltage activated Ca_V_s we have recorded, whereas the systolic phase would be mainly driven by high-voltage activated Ca_V_s. CHL elongates both systole and diastole in the intact heart, a result that matches its effect on both the low and the high-voltage activated components of current flowing through Ca_V_s. In the fly heart, RNAi repression of Ca_V_1 expression decreased both heart rate and intracellular calcium transients showing its strong involvement in cardiac function^33^. Since bee cardiomyocytes do have caffeine-sensitive intracellular Ca^2+^ stores, excitation–contraction coupling may involve both RyR and Ca_V_1. Heart contraction in the insect *T. molitor* is dependent on calcium stores as well, as demonstrated earlier with modulators of RyRs and SERCA pumps (sarcoplasmic/endoplasmic reticulum Ca^2+^-ATPase)^32^. In vertebrate cardiomyocytes, RyR and Ca_V_ interact and lead to a calcium-induced calcium release mechanism (CICR)^53^. CICR has been shown in bee skeletal muscle as well^47^. In the bee genome, the pore subunit of Ca_V_1, Ca_V_2 and Ca_V_3 channels are each encoded by a single gene^50^. None of alpha subunits from the three honeybee Ca_V_ families show a particularly high amino-acid homology with a specific mammalian subunit. For instance, the honeybee Ca_V_1 alpha subunit shows a similar homology (54–56%) with humans Ca_V_1.1, Ca_V_1.2, Ca_V_1.3. A single *ryr* gene is present in the bee genome (as in other insects) and the bee protein shares an amino acid identity of 45, 47 and 46% with the three mammalian subtypes (RyR1 the skeletal muscle type, RyR2 the cardiac muscle type and RyR3 an ubiquitous type), respectively^54–57^. Future studies will teach us to which extent the interplay between Ca_V_s and RyRs can participate in the autorhythmic activity of cardiomyocytes, in combination with other channels such as calcium-activated potassium channels.

In conclusion, cell electrophysiology and Ca^2+^ imaging on bee cardiomyocytes as well as measurements of the bee cardiac rhythm provide useful data on the effects of chlorantraniliprole in insects. This work brings new toxicological clues to better characterize the toxicity of diamides insecticides for useful insects.

## Methods

### Honey bees

Honey bees (*Apis mellifera*) were collected from the experimental apiary of the Abeilles & Environment Research Department located in Avignon. For electrophysiology, newly emerged bees (recognizable by specific anatomic and behavioural traits) were directly collected on combs with developing brood and placed in aerated plastic cages with 2 feeders containing water or commercial Candi sugar paste (Apifonda, Icko-Apiculture). For the *ex-vivo* study on heart, adult bees of undetermined age were collected on frames devoid of brood.

### Heart rate measurements

To characterize whole-heart activity, adult bees were cooled until lethargic immobility and then killed by decapitation. The abdomen was cut out from the body and pinned ventral side up with minuten pins on a Sylgard-coated Petri dish. Sternites were cut out and the cardiac tube in dorsal position was exposed by removing other internal organs (major tracheas, digestive tract, ventral nerve chord). Extreme care was taken to remove the hindgut and the venom sac without damage. The cardiac tube is a ~ 6 mm long translucent vessel composed of five chambers pumping hemolymph from the distal to the proximal part of abdomen, transporting nutrients to the thorax region via the aorta. After dissection, the heart remained attached to dorsal sternites via its alary muscles. The preparation was placed in a 3 mm deep pool (volume 0.5 ml) and continuously superfused with a control Tyrode solution containing 2 mM calcium (see Solutions) at a gravity-driven flow rate of 1.5 ml/min running from the distal to the proximal end of the heart. Liquid suction was made at the proximal end with a peristaltic pump. At 25 × magnification, under the annular LED light attached to the binocular’s condenser, the cardiac tube appeared white as compared with the surrounding tissue. Cardiac contractions were optically measured with a webcam mounted on the binocular and operated with the Webcam Capture plugin (written by Jérôme Mutterer) under the Image J software^58^. A glass coverslip was placed above the pool to prevent light variations that would arise from reflection on any liquid meniscus. A custom Image J macro allowed a real-time quantification of mean light intensity measured in images captured by the Webcam Capture plugin. During diastolic dilation, pixel intensity values were minimal, whereas during the systolic contraction phase, grey levels were maximal which allowed to track the contraction status and the heart rate.

In order to ensure a better exposure of hearts to the target CHL concentrations, gravity-driven perfusion of the control solution was stopped before adding the solution to the bath (500 µl added with a pipette, *i.e.* a volume equal the experimental well’s volume). After the incubation period (1 min), gravity-driven perfusion with control solution was turned back on, and a bath application of control solution (500 µl) was achieved to ensure the best wash out possible.

### Isolation of cardiomyocytes

The dorsal abdomen from newly emerged bees were prepared as in the *ex-vivo* study. They were bathed 20 min at 37 °C in a dissociation solution inspired from a successful procedure designed to isolate skeletal muscle fibers^47^. The dissociation solution was made of a calcium-free Tyrode, containing four enzymes: collagenase type 1, trypsin, papain, and pronase at concentrations 0.5, 1.5, 1.5 and 1 mg/ml, respectively. Whole hearts were rinsed twice, first in calcium-free in Tyrode’s solution without calcium first and then in control Tyrode (2 mM CaCl_2_). Whole hearts and surrounding tissues still attached to the cuticle were sucked out with a pipette and gently ejected in a Petri dish (Corning—430165) to disperse cardiomyocytes.

### Electrophysiology

Cardiomyocytes were monitored and imaged at × 100—× 400 magnification with a Qicam camera (QImaging Corp.) attached to the lateral port of a Leica DMIRB inverted microscope. A patch-clamp amplifier (RK400, Bio-Logic, Claix, France) was used in the voltage-clamp mode to measure membrane currents in the whole-cell configuration. Voltage pulse generation and data acquisition were done using WinWCP software (John Dempster, Strathclyde University, UK) driving an A/D, D/A converter (PCI-6014 board, National instruments Corp. Austin, TX, USA). Patch-clamp pipettes were pulled from borosilicate glass capillaries on a vertical pipette puller (P30, Sutter Instrument Co, Novato, AS, USA). Sylgard-coated electrodes were used to minimize the residual pipette capacitance that was analogically compensated for. The resistance of microelectrodes filled with internal solution (see Solutions) ranged between 2.4 and 3 MΩ in standard extracellular solutions. A micromanipulator (PCS 5200-2 Exfo Burleigh) was used to attach pipettes to cardiomyocytes cell membrane. In voltage-clamp mode, the microelectrode offset junction potential was nulled prior to seal formation. After seal formation, residual microelectrode capacitance was zeroed with a fast-analog compensation circuit available on the amplifier. Before reaching whole-cell configuration, the resting holding potential was set at -80 mV. After establishing the whole-cell configuration, series resistance was maximally compensated for. A period at least 5 min was respected in this configuration to ensure sufficient dialysis of cytoplasm with the intrapipette solution. Cell capacitance was calculated by integrating the linear capacitive currents obtained in response to a 10 ms pulse from − 80 to − 70 mV. To construct current–voltage (I/V) curves, cells were submitted to 400 ms voltage ramps from − 80 to + 40 mV followed by a 100 ms step depolarization from − 80 to 0 mV. Step depolarizations were also used to monitor currents kinetics. In order to subsequently subtract linear passive resistive and capacitive membrane currents, a P/4 protocol was used. Each test pulse was preceded by a series of 4 pulses of amplitude of one fourth in the opposite direction. Currents were low-pass filtered at 3 kHz with a filter available on the amplifier and sampled at 10 kHz. In the current-clamp mode, action potentials were recorded in response to current step injection of 1 s in order to depolarize the cells from a membrane potential set at -80 mV with a basal current injection. Cells were continuously perfused with a gravity-driven system made of polyethylene tubes (80 µl/min), with a tip aperture of 200 µm in diameter (dead volume ~ 15 µl). The perfusion tip was placed less than 200 µm away from the cell in order to ensure a rapid exposure of the cell membrane. All experiments were performed at room temperature (20–22 °C).

### Calcium imaging

For cytoplasmic calcium measurements, whole hearts (obtained after the same enzymatic treatment as for electrophysiology) were ejected into a glass-bottom dish (ibidi, Germany) containing a Tyrode solution (2 mM Ca^2+^). Cell were loaded for 30 min at room temperature with the cell permeant dye Fluo-8-AM (5 μM) and rinsed with Tyrode (2 mM). Fluo-8 loaded cardiomyocytes attaching to glass were observed at 40x (water immersion objective, HC PLAPO CS2, NA = 1.10) with a laser scanning confocal microscope (Leica, model SP8) operated with the software LAS X (Leica). Fluo-8 was excited with the 488 nm wavelength of an argon laser and the emitted fluorescent light was measured through a band-pass filter (505–570 nm) and digitized at 12 bits. Image series of 512 × 512 pixel (pixel size 0.45 µm) were captured at 1 Hz. Cells were exposed to CHL (0.01, 0.1 and 1 µM) with a gravity-driven perfusion system similar to the one used for patch-clamp experiments. In order to ensure that Ca^2+^ cytoplasmic fluctuations were originating from intracellular sources only, CHL was dissolved in Tyrode without Ca^2+^. Image series were processed with the Image J software (NIH). In each image series, mean fluorescence was measured in a region of interest onto the cell. Mean background fluorescence (measured in a zone devoid of cell) was subtracted from the cell fluorescence. Fluorescence variations were measured only in the absence of cell contraction and expressed as ΔF/F_0_, with F_0_ the resting fluorescence (baseline) and ΔF the change in fluorescence from baseline. ΔF/F_0_ were measured for amplitudes > 2SD above the mean resting level. The perfusion system had a dead volume of 15 µl for a perfusion rate of 90 µl/min. Vertical bars in Fig. 3 indicate the periods of perfusion with each CHL concentration taking into account a 10 s delay introduced by the dead volume.

### Solutions

The heart rate measurements were performed in a Tyrode solution containing 2 mM Ca^2+^. Standard extracellular solution for cardiomyocytes dissection contained (in mM): 140 NaCl, 5 KCl, 10 HEPES, 2 MgCl_2_ (pH 7.2), adjusted to pH 7.2 with NaOH (300 mOsm/l). CaCl_2_ 2mM was added to this solution in order to obtain a Tyrode 2Ca. Intracellular (pipette) solution contained (in mM): 140 K-gluconate, 2 MgCl_2_, 10 EGTA, 10 HEPES, pH 7.2 (292 mOsm/l). To record currents through Ca_V_s, the extracellular solution contained (in mM): 140 TEA-MeSO_3_, 2 BaCl_2_, 2 MgCl_2_, 1 4-amino-pyridine, 10 HEPES adjusted to pH 7.2 with MeSO_3_ (300 mOsm/l). A 10 mM nifedipine stock solution was prepared in DMSO and diluted to obtain 10 µM concentration and a final DMSO percentage of 0.1%. To match this DMSO concentration, control solutions were supplemented with DMSO 0.1%. A 500 mM NiCl_2_ stock solution was prepared in deionized water and diluted to final concentration 2 µM. Chlorantraniliprole 10 mM was prepared in DMSO and diluted to 10 µM. In Fig. 2 D, CHL was dissolved at 10 µM in Tyrode with Ca^2+^ (2 mM). In Fig. 3, CHL was dissolved at 0.01, 0.1 and 1 µM in a Tyrode without Ca^2+^. DMSO was always adjusted to 0.1%. All compounds were purchased from Sigma-Aldrich. The experimental CHL concentration range chosen is consistent with earlier in vitro and in vivo experiments on CHL and other anthranilic diamides^41,42,46^.

### Statistical analysis

Statistics were performed under OriginPro software (OriginLab, USA) and Graphpad Prism. Data were tested for normality using the Shapiro–Wilk test. Non-parametric Wilcoxon signed-rank test or Mann–Whitney tests were used to compare distributions of ranks when appropriate. Groups were considered different for P < 0.05. To compare multiple groups, a Kruskall-Wallis anova was used, followed by a post-hoc Dunn’s test. Data are presented as mean ± S.E.M (standard error of the mean).

### Supplementary Information

Supplementary Figure S1.

## Data Availability

All data generated or analysed during this study are included in this published article.
