# Supplementary material for: Cardiotoxicity of the diamide insecticide chlorantraniliprole in the intact heart and in isolated cardiomyocytes from the honey bee
Source: Sci Rep. 2024 Jun 28;14:14938. doi: 10.1038/s41598-024-65007-2 (PMC11213956; doi:10.1038/s41598-024-65007-2)

Cardiotoxicity of the diamide insecticide chlorantraniliprole in the intact heart and in isolated cardiomyocytes from the honey bee.

Mahira KAABECHE, Mercedes CHARRETON, Aklesso KADALA, Jérôme MUTTERER, Pierre CHARNET and Claude COLLET

Supplementary Figure S1. Effect of nifedipine on the high-voltage activated Ba^{2+} current during a step depolarization from -80 to 0 mV.

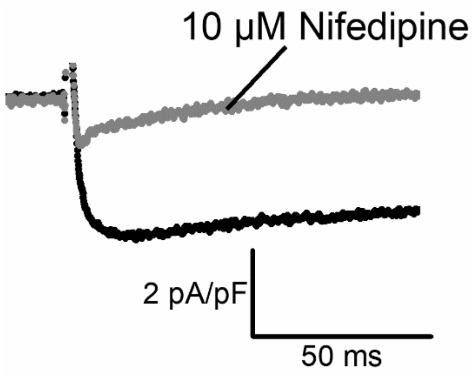

Supplement: Supplementary file 1 — Supplementary Figure S1. [file 41598_2024_65007_MOESM1_ESM.pdf]
